# Optimizing Silanization to Functionalize Stainless Steel Wire: Towards Breast Cancer Stem Cell Isolation

**DOI:** 10.3390/ma13173693

**Published:** 2020-08-21

**Authors:** Aliya Bekmurzayeva, Kanat Dukenbayev, Helena S. Azevedo, Enrico Marsili, Daniele Tosi, Damira Kanayeva

**Affiliations:** 1 Science, Engineering and Technology Program, Nazarbayev University, Nur-Sultan 010000, Kazakhstan; abekmurzayeva@nu.edu.kz; 2 National Laboratory Astana, Nazarbayev University, Nur-Sultan 010000, Kazakhstan; daniele.tosi@nu.edu.kz; 3 School of Engineering and Digital Sciences, Nazarbayev University, Nur-Sultan 010000, Kazakhstan; kdukenbayev@nu.edu.kz (K.D.); enrico.marsili@nu.edu.kz (E.M.); 4 School of Engineering and Materials Science, Queen Mary University of London, London E1 4NS, UK; h.azevedo@qmul.ac.uk; 5 School of Sciences and Humanities, Nazarbayev University, Nur-Sultan 010000, Kazakhstan

**Keywords:** stainless steel wire, functionalization, silanization, aptamers, breast cancer stem cells

## Abstract

Chemically modified metal surfaces have been used to recognize and capture specific cell types and biomolecules. In this work, stainless steel wires were functionalized with aptamers against breast cancer stem cell markers. Stainless steel wires were first electropolished and silanized via electrodeposition. Aptamers were then attached to the silanized surface through a cross-linker. The functionalized wires were able to capture the target cells in an in vitro test. During surface modification steps, wires were analyzed by atomic force microscopy, cyclic voltammetry, scanning electron and fluorescence microscopy to determine their surface composition and morphology. Optimized conditions of silanization (applied potential, solution pH, heat treatment temperature) for obtaining an aptamer-functionalized wire were determined in this work together with the use of several surface characterization techniques suitable for small-sized and circular wires. These modified wires have potential applications for the in vivo capture of target cells in blood flow, since their small size allows their insertion as standard guidewires in biomedical devices.

## 1. Introduction

Stainless steel (SS) is commonly used in biomedical devices and applications because of its biocompatibility and mechanical properties [1,2]. Recent applications of SS include functionalized guidewire for the capture of cells [3], biosensing [4] and sorptive extraction of contaminants [5]. All of these applications require chemical modification of the surface. Silanization of the SS surface is commonly adopted as a preliminary step prior to functionalization with specific biomolecules. APTES ((3-aminopropyl)triethoxysilane) is a widely used silane-coupling agent in silanization. The quality of the silanization process plays an important role in further surface functionalization [6], requiring a thorough investigation of silanization conditions.

With the recent progress in both treatment and prognosis of breast cancer, recurrence and resistance to therapy still remain the biggest problems in managing breast cancer [7] and metastasis, being the main reason for the death of patients. Several studies suggest that only a small subset of tumor cells, called breast cancer stem cells (BCSC), is responsible for increased resistance to therapy, and that these cells drive tumor growth and development. BSCS are similar to normal stem cells, as they are capable of self-renewal and multi-lineage differentiation [8,9]. Similar to CSC, circulating tumor cells (CTC) occur at low concentration in the blood circulation, making their capture challenging. To overcome this problem, the CellCollector™ device, which is based on a SS medical guidewire coated with epithelial cell adhesion molecule (EpCAM) antibodies, was developed [10]. The device showed a superior performance compared with CellSearch™, a device cleared by the Federal Drug Administration of the United States for CTC detection [10]. CellCollector™ was tested in patients with breast [11,12,13], prostate [14,15], lung [16,17,18], head and neck cancer [13]. It is estimated that by putting the functionalized wire in contact with patients’ blood for 30 min, it is exposed to 1.5–3 L of blood [19], and this improves the chances of capturing rare events such as binding of CTCs in the blood. Using this guidewire, a decrease in the CTC count was measured after treatment (surgery) and more CTCs were found in later stages of disease [20], making it useful for monitoring treatment responses. Furthermore, capturing CTCs using this device and further molecular characterization of the cells could bring personalized medicine closer to reality [12,21]. Other devices designed to isolate CTCs from the blood stream include flexible magnetic wires that capture cells previously labelled with injected magnetic nanoparticles [22] and a “cytosensor” based on the functionalized SS needle (also covered with EpCAM antibodies) [4]. All of these studies, however, rely on the detection of CTC by EpCAM antibodies. EpCAM can be reduced in its expression during epithelial-to-mesenchymal transition, which is associated with dissemination of tumor cells [23]. Thus, there is a need for the discovery of more specific markers and/or a combination of methods to isolate tumor cells with stem cell-like properties (i.e., CSC) [24].

**Figure 1:**
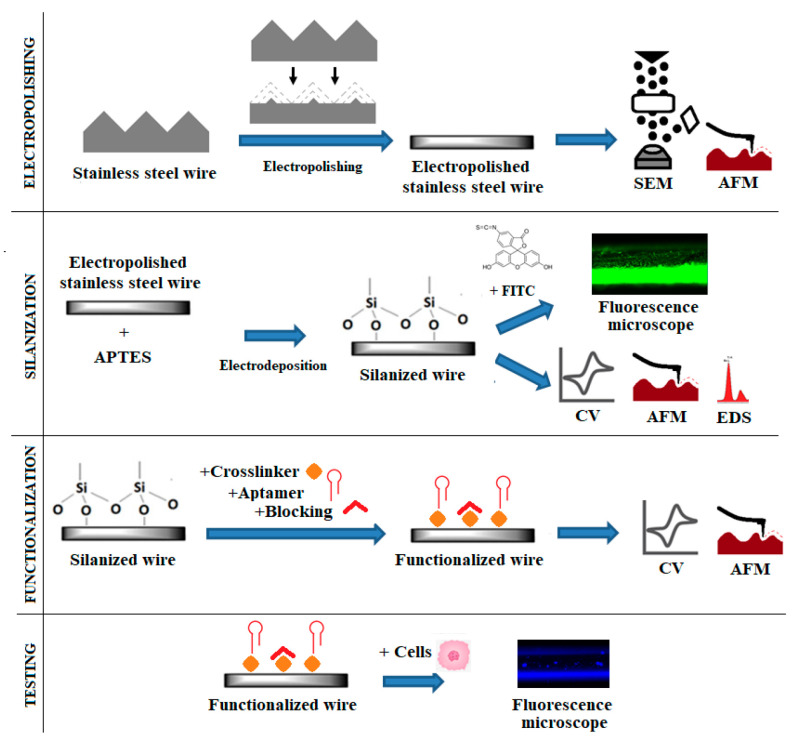
Overview of the functionalization of the stainless steel (SS) wire to capture breast cancer stem cells (BCSC). APTES: (3-aminopropyl)triethoxysilane; AFM: atomic force microscopy; CV: cyclic voltammetry; FITC: fluorescein isothiocyanate; EDS: energy-dispersive X-ray spectroscopy; SEM: scanning electron microscopy.

This work aimed to optimize surface pre-treatment and the subsequent silanization of SS wires via electrodeposition. After being covered with APTES, the SS wires were further functionalized with a cross-linker and aptamers against BCSC biomarkers and tested to capture the target cells, BCSC. The surface and the in vitro BCSC capture were characterized using several surface characterization methods. The overall scheme of the work undertaken in this study is shown in Figure 1. The work consisted of several stages: electropolishing of the wire, optimization of silanization through electrodeposition, functionalization of the surface with aptamers, and testing it to capture target cells. After each of the surface modifications, the surface was analyzed by surface characterization techniques, as shown in Figure 1.

## 2. Materials and Methods

### 2.1. Pre-Treatment of SS

The 316L SS wire (diameter 0.18 mm; The Crazy Wire Company, Warrington, UK) was cut into pieces (ca. 7–8 cm) forming a hook on one end. One of two pretreatment methods was applied: (i) sonication only (sample: sonic) and (ii) electropolishing (sonication and further electropolishing) (sample: Elpol (electropolished)). Sonication was conducted in an ultrasonic bath (Branson 1800, Danbury, CT, USA) according to [25], where the samples were sonicated in deionized water, acetone, ethanol with an additional sonication in water (each for 10 min). Electropolishing was performed in a two-electrode setup using PalmSens4 potentiostat (PalmSens BV, Houten, The Netherlands), where flat SS was used as a cathode and the applied 1.8 V potential obtained a current of ca. 30 mA when 50–55 mm of the anode was immersed in the electrolyte. Electropolishing was conducted under constant temperature (75–80 °C) in a solution of 11 M H_3_PO_4_ and 4.5 M H_2_SO_4_ [26] with a constant distance between electrodes (ca. 15 mm) for 300 s.

### 2.2. Electrodeposition of APTES

Silane was electrodeposited on wires in a three-electrode system consisting of platinum (Pt) wire as a counter electrode, silver (Ag) wire as a quasi-reference electrode (QRE) and pretreated samples as working electrodes on an IM6 electrochemical station (ZAHNER-Elektrik, Kronach, Germany). Ag electrode was coated with Ag/AgCl ink (ALS Co., ltd, Tokyo, Japan) and heated at 70 °C for 20 min in an oven before use. Electrodeposition solution contained 0.02 M APTES (Sigma Aldrich, Steinheim, Germany), to which different volumes of 0.1 M HCl and ethanol were added (to obtain a pH of 4–6) for a final volume of 131 mL. Constant negative potential (−0.6; −0.8; −1.0 or −1.2 V vs. Ag QRE) was applied to the samples for 30 min, and they were heat treated for 1 h at 130 °C. After heat treatment, the wires were rinsed in water for 2 min to remove physiosorbed molecules [27] (sample: HT (heat treated after APTES)) and cut for further analyses.

### 2.3. Cyclic Voltammetry

Cyclic voltammetry (CV) was performed in phosphate-buffered saline (PBS) (pH 7.4) containing 0.1 M KCl and 1 mM K_3_[Fe(CN)_6_]/K_4_[Fe(CN)_6_] with a potential set of from −0.6 V to 0.7 V and a scan rate of 50 mV/s. The three-electrode system consisting of Pt wire as a counter electrode, an Ag/AgCl (3 M NaCl) reference electrode (RE) (Zimmer and Peacock, Horten, Norway) and a working electrode (sample of interest) was used on the IM6 electrochemical station. Thirteen CV cycles were performed, and the last one was used for comparison.

### 2.4. Scanning Electron Microscopy (SEM) and Energy-Dispersive X-ray Spectroscopy (EDS) Analysis

The samples were analyzed on FeSEM Auriga (Crossbeam 540, ZEISS, Oberkochen, Germany) for obtaining SEM images at 5 kV at magnifications of 500 and 2000 times. For EDS analysis, a JSM-IT200 (JEOL, Tokyo, Japan) microscope at 20 kV at a magnification of 2000 times was used. The samples (sonic, Elpol, HT) were attached onto a carbon tape (Agar scientific, Stansted, UK) before analysis.

### 2.5. FITC Analysis

The HT samples were incubated with 125 µg/mL fluorescein-5-isothiocyanate (FITC) (Sigma Aldrich, Steinheim, Germany) in sodium carbonate/bicarbonate (pH 9.2) buffer for 2 h in the dark and washed with ethanol for 5 min and dried. Elpol (no silanization) served as a control sample. The samples were then attached on glass slides and visualized on a fluorescence microscope (FLoid™ cell imaging station, Thermofisher, Loughborough, UK).

### 2.6. Immobilizing Ligands

To immobilize ligands on silanized wires, the HT samples were incubated with a cross-linker (5% glutaraldehyde (GA) in PBS) for 1 h and rinsed with PBS (sample: HT-GA (heat treated after APTES-glutaraldehyde)). To immobilize the aptamers, the HT-GA samples were incubated with amine-modified CD44-aptamers (5′-[AmC6F]-CCAAGGCCTGCAAGGGAACCAAGG-3′; Sigma Aldrich, Steinheim, Germany), which were originally selected by systematic evolution of ligands by exponential enrichment (SELEX) [28] and then truncated in an in silico study [29]. For immobilization, cross-linked wires were incubated with 2.5 µM of aptamers in binding buffer (BB; 50 mM Tris-HCl and 20 mM MgCl_2_, pH 7.4) for 1 h at 37 °C and rinsed with BB (sample: HT-GA-Apt (heat treated after APTES-glutaraldehyde-aptamer)). Unreacted aldehyde groups on the samples were blocked by ethanolamine (0.5 M) for 15 min and rinsed with BB. After blocking, aptamer-functionalized samples (sample: HT-GA-Apt-block or functionalized wire) were rinsed with washing buffer (BB with 0.1% Tween 20) and stored at 4 °C in BB before further use. Electropolished wire (no silanization) was used in all functionalization steps and treated as control samples (sample: Elpol-GA-Apt-Block or control wire).

### 2.7. Atomic Force Microscopy (AFM)

High-resolution topographical characterization of the surfaces was carried out using SmartSPM 1000 (AIST-NT Inc., Novato, CA, USA) in AC (alternating current) mode (non-contact mode). All measurements were performed with a scanning rate of 0.7 Hz; the scan range was 5 µm × 5 µm (2.5 µm × 2.5 µm or 1 µm × 1 µm) in X-Y; and height Z was set automatically. Super sharp type “NSG30_SS” cantilever (Golden Silicon Probes; TipsNano, Tallinn, Estonia), with a tip radius curvature of up to 2 nm, a force constant of 22–100 N/m, and a resonance frequency of 240–440 kHz in air, was used.

### 2.8. Testing the Functionalized SS Surface to Capture BCSC

Cells from a well-characterized human breast cancer stem cell line (Celprogen; Torrence, CA, USA, Cat. no. 36102-29) were grown on a T75 flask coated with a human breast cancer stem cell extracellular matrix (ECM) (Celprogen; Cat. no. E36102-29) in human breast cancer stem cell complete media with serum (Celprogen; Cat. no. M36102-29PS). Cells were dissociated with 2.5 mM PBS-EDTA (ethylene diaminetetraacetic acid; Thermofisher, Loughborough, UK) for 5 min, washed with PBS and filtered through a cell strainer (70 µm). Cells (5 × 10^5^ cells/mL) were incubated with functionalized and control samples for 30 min and then fixed with 3.7% formaldehyde (Sigma Aldrich, Steinheim, Germany) for 15 min and stained with DAPI (4′,6-diamidino-2-phenylindole). Wires were then visualized on a fluorescence microscope (FLoid™ cell imaging station, Thermofisher, Loughborough, UK).

## 3. Results and Discussion

### 3.1. Pre-Treatment of SS

**Figure 2:**
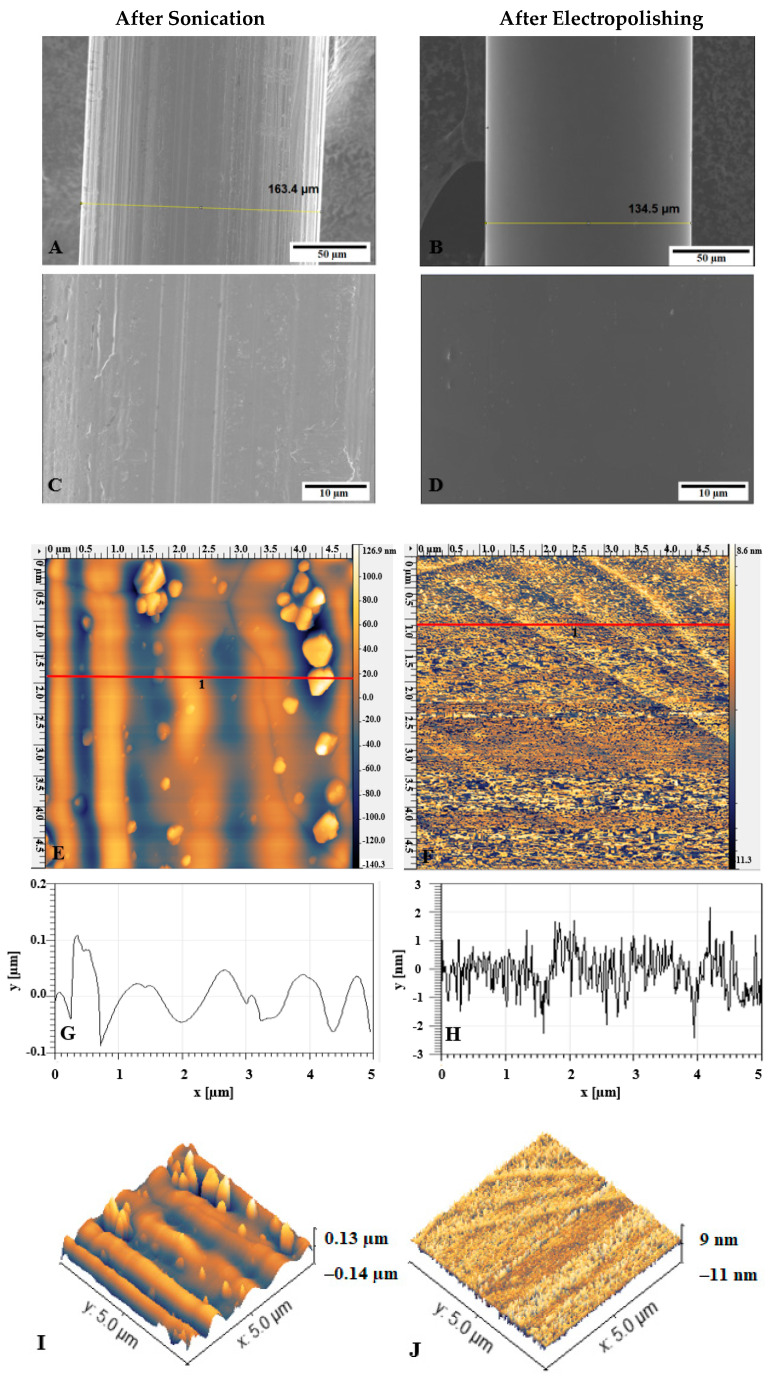
SEM and AFM micrographs of SS wire after sonication (**A**,**C**,**E**) and after electropolishing (**B**,**D**,**F**); (**E**,**F**) 5 µm × 5 µm AFM images of wires; (**G**,**H**) profiles of (**E**) and (**F**) respectively along the line; (**I**,**J**) corresponding 3D images of (**E**) and (**F**).

The SS wires (a diameter of ~160 µm as measured by SEM) were electropolished for 300 s at 30 mA before silanization. The electropolished SS wires were then analyzed with AFM and SEM. The wire diameter decreased to ~135 µm, and the surface appeared well polished (Figure 2A–D). Electropolishing for 100 s and 200 s was not enough to produce a fully polished surface (Supplementary Figure S1). Examining samples after electropolishing with AFM further supported the results from SEM of successful electropolishing, as seen from Figure 2E–H. Large unevenness of the surface levelled out after electropolishing.

### 3.2. Electrodeposition of APTES: General Consideration

Before silanization, the substrate needs to be hydrolyzed to allow sufficient silanol groups (Si-OH) to react with the surface [30]. During electrodeposition of silane, applied negative potential causes the formation of hydroxyl ions that catalyze the binding of silane on the surface [31]. The electrodeposition of silane-coupling agents offers a more uniform coating [32] and the possibility of controlling thickness and geometrically coating complex shapes [33]. By using electrodeposition, it is possible to avoid additional steps in surface modification (surface hydroxyl generation). Characterization of the functionalized wire after silanization and further with ligands poses some challenges since common techniques such as contact angle measurement and Fourier-transform infrared spectroscopy (FTIR) cannot be used due to the small size of the wire. Therefore, other techniques, such as AFM, EDS, CV, and FITC analysis, were exploited in this work.

### 3.3. Electrodeposition of APTES: Applied Potential

**Figure 3:**
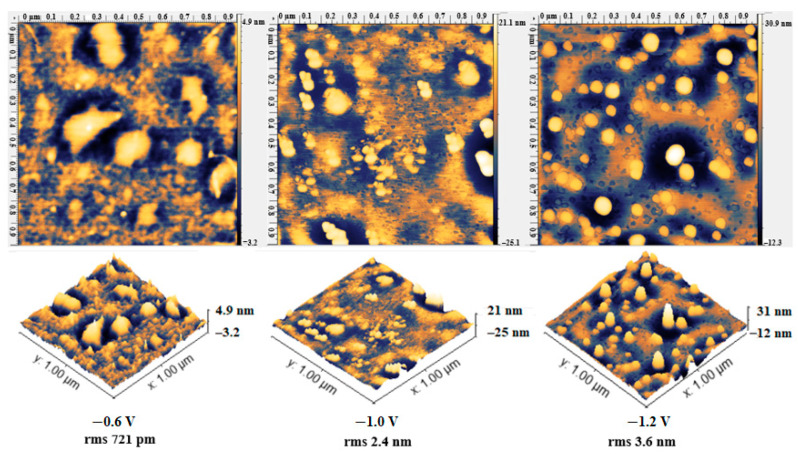
AFM micrographs of electropolished SS wires after electrodeposition of APTES under different potentials; 1 µm × 1 µm images and their 3D images and rms are shown. Samples at −0.8 V are shown in Figure 4, and the 1 µm × 1 µm images for each applied potential and their 3D images are shown.

**Figure 4:**
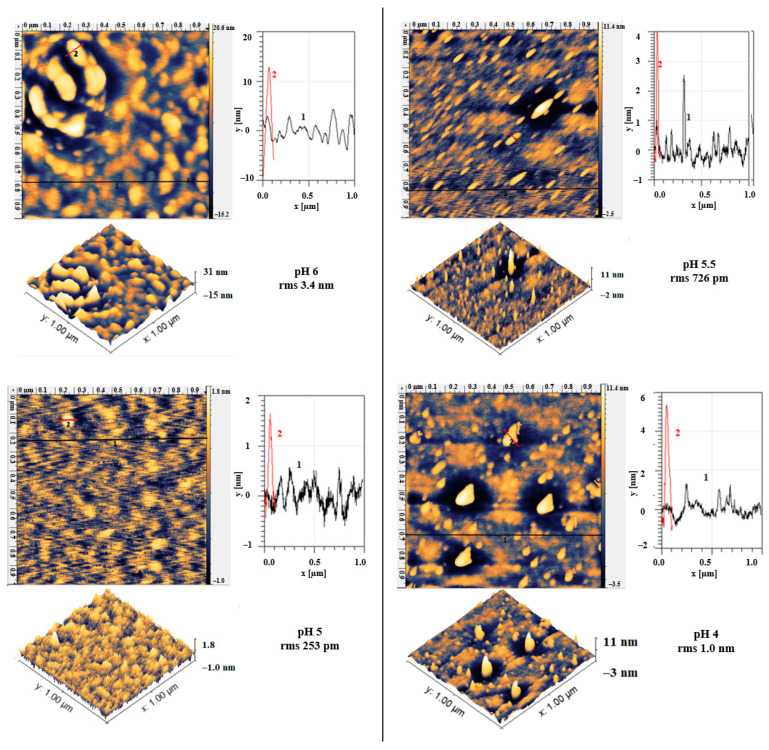
AFM micrographs of electropolished SS wires after electrodeposition of APTES at the applied potential of −0.8 V for 30 min in solutions with pH values of 6; 5.5; 5, and 4. For each pH value, 1 µm × 1 µm images, their 3D images and profiles along the lines are shown.

Electrodeposition using different applied potentials was studied on two different SS samples: sonicated (sonic) and electropolished (Elpol). Sonication did not change the surface roughness of the samples, thus, the AFM images after electrodeposition at −0.8, −1.0, and −1.2 V were not distinguishable from the samples prior to electrodeposition (Figure S2). On the other hand, electropolishing results in a smooth sample surface, which allowed accurate AFM characterization of the silanized surface. APTES electrodeposition on electropolished SS wires under different potentials produced surfaces with different morphology when studied on AFM (Figure 3). With the increase in the applied potential (from −0.6 to −1.2 V), the roughness of the surface increased, as shown by the increase in the root mean square roughness (rms) of the surfaces. The increase in film thickness with more negative potentials was also reported during silanization of SS with two different silanization agents [33], where at higher potential irregular films were formed, probably due to hydrogen evolution. The sample silanized at −1.2 V in this study also had more polymerized particles. The potential of −0.8 V (Figure 4) was further studied since it produced more uniform surfaces and the chronoamperometry graphs (Figure S3) were decreasing and then stabilizing, as previously reported for silanized poly-ethylene glycol on SS [23]. In the literature, a monolayer of APTES with a height of 0.8 ± 0.1 nm was reported to produce a well-functionalized film, while higher values were considered polymerized APTES (more than one layer) [6]. Other studies report higher particle height of 1.8 nm [34] and less than 2 nm for a single APTES layer [35]. Howarter et al. [36] obtained surfaces with rms values of 1.5–2.9 nm corresponding to 2–4 APTES layers with no agglomerates or multi-island growth present on the surface. Rms value of 3–4 nm were also reported in [37]. In light of these previous studies, it was decided to consider APTES functional if the layer uniformly covered the samples and the range of particle size was 0.8–2.0 nm, with a few larger particles (indicating polymerized APTES) on the surface. Surfaces with a particle height of less than 0.8 nm were not considered silanized.

CV can be used as a surface characterization technique, as the changes in peak current and peak-to-peak separation after each modification step correlate with the electron transfer resistance of the surface, which in turn indicates the formation of a non-conductive silanized layer [38]. The CV of wires silanized with APTES using different applied potentials is shown in Figure S4. The electron transfer resistance followed the following order: −1.2 V < Elpol < −0.6 V < −0.8 V, with −0.8 V producing the optimal silanized layer. The lower electron transfer resistance at −1.2 V is likely due to a less uniform coverage of the surface and formation of larger particles, as shown on AFM (Figure 3). The samples silanized at −0.8 V showed the highest concentration of Si, C, and O in the EDS analysis and were further functionalized with aptamers (Figure S5).

### 3.4. Electrodeposition of APTES: pH of the Solution

Together with the applied potential during electrodeposition of APTES, different pH values of the electrodeposition solution (pH 4–6) were studied. AFM images of these silanized surfaces are shown in Figure 4. All the surfaces appear covered with 0.8–1 nm particles. Some larger particles could also be seen for pH 4 (>6 nm) and pH 6 (>10 nm). At pH 5.5 some particles with 4 nm size were formed. Solution pH is an important factor during electrodeposition; it affects hydrolysis and condensation of APTES. Usually, before being immobilized on metal, it has to be hydrolyzed to form silanol (Si-OH) groups. However, after hydrolysis, condensation can take place where it is polymerized and precipitated [30], thus, it is necessary to minimize this process. It was reported by Tesoro et al. [39] that at a pH of 7, the hydrolysis rate is minimal and at a pH of 4.3, condensation is minimum for APTES, where condensation is a base-catalyzed reaction. Since reducing silane concentration also minimizes its condensation [30], its APTES concentration was also reduced in this work.

**Figure 5:**
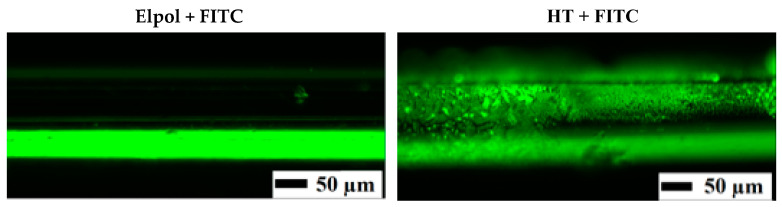
FITC analysis of the control (Elpol-electropolished) and silanized samples (HT) (electrodeposition at the applied potential of −0.8 V for 30 min) as visualized by fluorescence microscopy.

The presence of APTES on the surface was also investigated by incubating with FITC. FITC is a dye with an N=C=S functional group which reacts with amine and thiol groups [40]. Thus, it can be used for fast qualitative assay of showing that the surface has been aminated with APTES. As seen in Figure 5, the control sample (Elpol + FITC) had lower signal intensity (autofluorescence) than the silanized samples (HT + FITC). Similarly, FITC analysis was used for qualitative analysis of various surfaces after silanization with APTES, including silicon oxide [40], poly(dimethylsiloxane) [41], nanoparticles [42], and titanium [43].

Silanized surfaces were analyzed by SEM/EDS. For silanized samples, that also showed blocking in CV, an increase in Si (from to 0.70 to 0.83 at%) when compared to Elpol was observed. Increased content of C (from 0 to 13.70 at%) and O (from 0 to 3.77 at%) was also observed for some samples. The presence of APTES on surfaces reported in the literature also showed an increase in elements such as C, Si, O [44,45,46]. SEM/EDS is also a widely used method for surface characterization. According to the literature, when surfaces (SS or other metals) silanized with APTES are visualized on SEM, they are either smooth [33] or have some aggregates (particle size not reported but estimated to be 10–60 nm) [27,47]. As opposed to SEM, EDS seems a more useful technique to study the surface after silanization.

### 3.5. Electrodeposition of APTES: Heat Treatment Temperature

**Figure 6:**
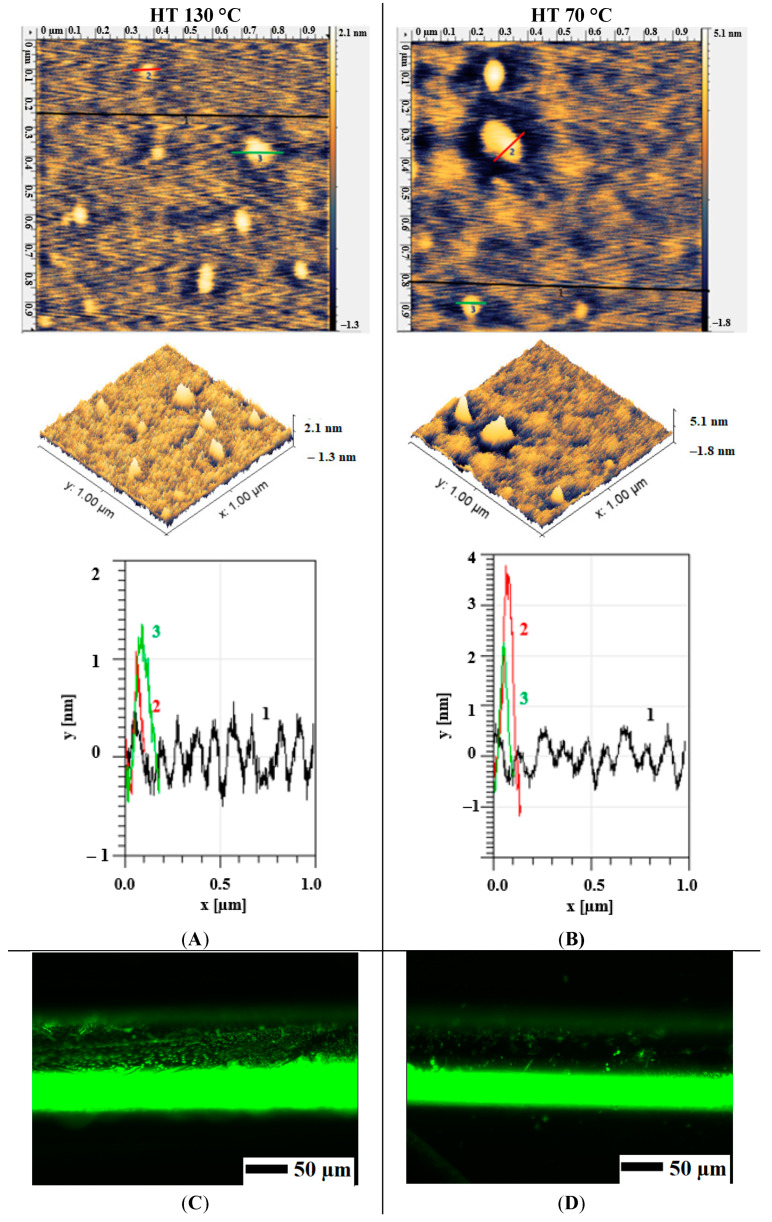
AFM micrographs (**A**,**B**) and FITC analysis (**C**,**D**) of SS wire at two different heat treatment temperatures (130 °C and 70 °C) after electrodeposition of APTES using −0.8 V vs. QRE.

Heat treatment after silanization is another important parameter to consider since it affects the covalent bond forming between APTES molecule and the surface. Two heat treatment temperatures (70 °C [4] and 130 °C [27]) were tested for HT after electrodeposition (Figure 6A,B) taken as the lowest and the highest reported in the literature. As a result, it was observed that at 130 °C, a more uniform layer was formed, while for 70 °C, some larger particles (up to 4.5 nm) were seen. Additionally, FITC analysis of samples after silanization and subsequent heat treatment at two different temperatures showed that at 130 °C the surface had a more uniform and higher signal for FITC than that of 70 °C (Figure 6C,D).

### 3.6. Attaching Aptamers

**Figure 7:**
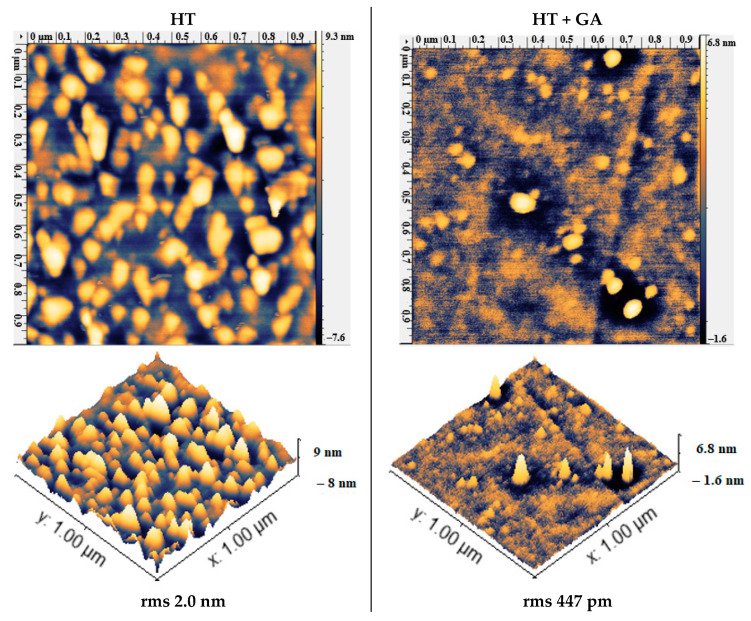
AFM micrographs of silanized SS before and after treatment with GA.

**Figure 8:**
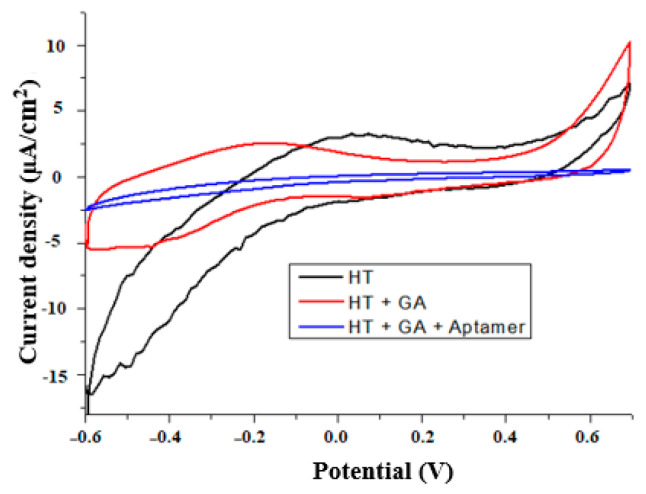
Cyclic voltammograms of silanized SS electrode (HT) after treatment with GA and aptamers. Potential vs. RE (3 M NaCl).

In order to immobilize ligands on a silanized surface, it has to be cross-linked first; in our case, we used GA whose aldehyde groups (-CHO) could be used to form imines for further immobilization [48]. In this work, we observed a decrease in the surface roughness after GA (Figure 7) with lowered rms from 0.1 to 2 nm across the samples. After treating with GA, it is expected that the surface either retains the same rms as the silanized surface (0.18 nm) [49] or becomes smoother, as reported by two studies (from rms 0.830 µm to 0.524 µm in [50] and rms from 0.69 to 0.51 nm in [35]). Binding of GA was also confirmed by CV where after GA, treatment of the HT sample lowered the peak current and increased the peak-to-peak separation (Figure 8). In the literature, when treated with GA, the APTES-silanized electrode [51] also had a lowered peak current and an increased peak-to-peak separation. This was attributed to the insulating film of GA forming on the surface and electrostatic repulsion between GA and the redox couple.

**Figure 9:**
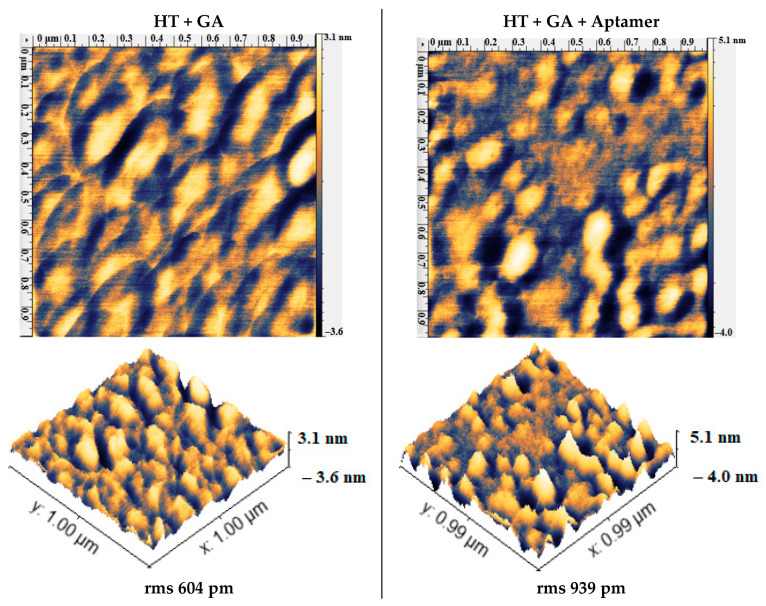
AFM micrographs of cross-linked SS after silanization (HT + GA) and after treatment with aptamers (HT + GA + aptamer).

After treatment with the cross-linker, the wire was incubated with aminated aptamers. In AFM, the aptamer-treated surfaces showed smoother (mean roughness Ra from 1.46 for the bare surface to 1.36 nm for the aptamer surface) [52] or rougher (1.79 nm for the bare surface to 2.53 nm for the aptamer surface) [53] surfaces in two different studies. We also observed a range of different results: smoother, rougher, or similar in surface topography, but all of them showed that the surface was modified from a previous HT-GA sample used in the reaction (Figure 9). Aptamer immobilization was also confirmed by CV. The aptamer-immobilized surface had a lowered current (Figure 8), suggesting blocking of the electron transfer between the redox couple and the surface by the aptamer layer. The control surface (Elpol not silanized) (Figure S5) had cyclic voltammograms that were not changed as much in all functionalization steps, suggesting that GA and the aptamer were immobilized on the silanized surfaces. The study of aptamers on electrodes with CV by [54] showed a lowering of peak current and a lowered peak-to-peak separation [55].

### 3.7. Testing the Surface to Capture Cells

**Figure 10:**
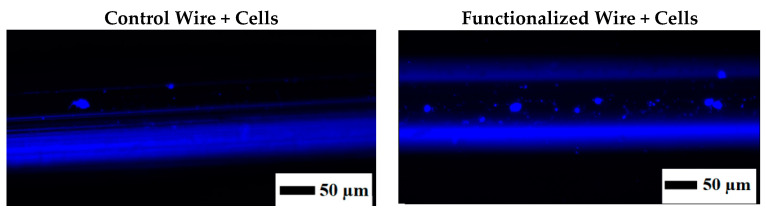
Ability of functionalized wires to capture target cells (breast cancer stem cells) as observed by fluorescent microscopy. Images of the control and functionalized wires after DAPI staining.

After being fully functionalized, the wires (together with a control wire) were tested to capture target cells. For this, BCSC were chosen as target cells given their importance in breast cancer. Well-characterized cells from patients with triple-negative breast cancer with a number of biomarkers and characteristics pertaining to BCSC were chosen in this study. This includes surface biomarkers such as CD133, CD44, SSEA3/4, Oct4, and important enzymes (alkaline phosphatase, aldehyde dehydrogenase, and telomerase) and tumorigenicity of less than 1000 cells. SS samples were incubated with the cells dissociated with PBS/EDTA. Using non-enzymatic cell dissociation was important for retaining cell surface proteins for further binding to the aptamers [56]. Figure 10 shows the results of fluorescence imaging after incubation with the cells. The functionalized wire was able to capture cells on the surface, as seen from blue-stained nuclei, while the control wire had a very low number of cells as compared to the functionalized wire. Performing this test was important to show that wires made of biocompatible material can capture specific target cells of interest. To further exploit the potential of the aptamer-functionalized wires for in vivo applications, this in vitro study should be replicated in a model that mimics blood flow (e.g., vein diameter, blood flow, and more complex media containing other cells and proteins). In the future, the functionalized wire also needs to be tested using control cells with no or low expression of CD44 biomarkers (such as breast epithelial cells) and tested with different cell concentrations.

## 4. Conclusions

Functionalization of metals is important in the fabrication of analyte-capturing metal substrates among other biomedical applications. Surface pre-treatment was a crucial first step in producing functional surfaces. Smooth surfaces were obtained after electropolishing, which not only improved silanization but also allowed their characterization by AFM after silanization. Having an additional tool to characterize the surface was important since the physical dimensions of the wire limited the use of other methods, such as contact angle measurement or FTIR. Silanization by electrodeposition was optimized as the first step before biomolecule-attaching on the wire. Based on the surface analysis techniques used, the best applied potential during electrodeposition was −0.8 V, and the pH of the solution was 5–5.5. Heat treatment of silanized wires at 130 °C produced better APTES-covered surfaces than at 70 °C, as shown both by AFM and FITC analyses. The functionalized wire was able to capture BCSC as opposed to the control wire when visualized by fluorescence microscopy. When the wire was characterized by the combination of methods (AFM, CV, EDS, and FITC analysis), a better overall view of the surface modification was generated. Small-diameter wires, with the ability to fit standard catheters and of being covered with aptamers against BCSC biomarkers could be important in diagnostics. After further assessment, they could be used in vivo to capture rare cancerous cells such as BCSC by processing a large volume of blood.

## Supplementary Materials

The following are available online at https://www.mdpi.com/1996-1944/13/17/3693/s1, Figure S1: Scanning electron micrographs of the wire after electropolishing for 100 and 200 s. Figure S2: AFM images of APTES electrodeposition on sonicated SS (sonic) wires under different potentials (10 µm × 10 µm and their corresponding 3D images). Figure S3: Chronoamperometry response for APTES electrodeposition on SS wires at −0.8 V for 30 min (potential vs. QRE). Figure S4: Cyclic voltammograms of electropolished SS electrode (Elpol) and silanized with APTES using different applied potentials (HT): −1.2; −0.8, and −0.6 V; CV conducted in PBS with a pH of 7.4 containing 0.10 M KCl and 1.0 mM [Fe(CN)_6_]^3−/4−^ with a scan rate of 50 mV/s. Potential vs. RE (3 M NaCl). Figure S5: Cyclic voltammograms of functionalizing steps of a control sample (Elpol—electropolished) with aptamers. CV conducted in PBS with a pH of 7.4 containing 0.10 M KCl and 1.0 mM [Fe(CN)_6_]^3−/4−^ with a scan rate of 50 mV/s. Potential vs. RE (3 M NaCl).

## References

[1] Zhang H., Han J., Sun Y., Huang Y., Zhou M. (2015). MC3T3-E1 cell response to stainless steel 316L with different surface treatments. Mater. Sci. Eng. C Mater. Biol. Appl..

[2] Hermawan H., Ramdan D., Djuansjah J., Fazel-Rezai R. (2011). Metals for biomedical applications. Biomedical Engineering—From Theory to Applications.

[3] Saucedo-Zeni N., Mewes S., Niestroj R., Gasiorowski L., Murawa D., Nowaczyk P., Tomasi T., Weber E., Dworacki G., Morgenthaler N.G. (2012). A novel method for the in vivo isolation of circulating tumor cells from peripheral blood of cancer patients using a functionalized and structured medical wire. Int. J. Oncol..

[4] Weng W.-H., Ho I.-L., Pang C.-C., Pang S.-N., Pan T.-M., Leung W.-H. (2018). Real-time circulating tumor cells detection via highly sensitive needle-like cytosensor-demonstrated by a blood flow simulation. Biosens. Bioelectron..

[5] Amiri A., Ghaemi F. (2017). Graphene grown on stainless steel mesh as a highly efficient sorbent for sorptive microextraction of polycyclic aromatic hydrocarbons from water samples. Anal. Chim. Acta.

[6] Libertino S., Giannazzo F., Aiello V., Scandurra A., Sinatra F., Renis M., Fichera M. (2008). XPS and AFM characterization of the enzyme glucose oxidase immobilized on SiO_2_ surfaces. Langmuir.

[7] Islam F., Gopalan V., Smith R., Lam A. (2015). Translational potential of cancer stem cells: A review of the detection of cancer stem cells and their roles in cancer recurrence and cancer treatment. Exp. Cell Res..

[8] Ribatti D. (2012). Cancer stem cells and tumor angiogenesis. Cancer Lett..

[9] Sampieri K., Fodde R. (2012). Cancer stem cells and metastasis. Semin. Cancer Biol..

[10] Nowaczyk P., Dlugaszewska S., Herold S., Krahn T., Mayer M., Morgenthaler N., Zabel M., Luecke K., Murawa D. A Novel Technology for In Vivo Isolation of circulating Tumor Cells in Breast Cancer Patients. Proceedings of the 9th European Breast Cancer Conference.

[11] Heyden A., Tomasi T., Zeni N., Herold S., Nowaczyk P., Schmitz A., Krahn T., Zabel M., Murawa D., Luecke K. (2011). In vivo isolation of circulating tumor cells. Eur. J. Cancer.

[12] Nowaczyk P., Herold S., Kim P.S., Schmitz A., Polom K., Murawa P., Morgenthaler N.G., Zabel M., Luecke K., Murawa D. (2012). Functionalized and Structured Medical Wire as a Device for In-Vivo Isolation of Circulating Tumor Cells in Breast Cancer Patients. Eur. J. Cancer.

[13] Li J., Geng C., Yan M., Wang Y., Ouyang Q., Yin Y., Wu L., He J., Jiang Z. (2017). Circulating tumor cells in patients with breast cancer were detected by a novel device: A multicenter clinical trial in China. Natl. Med. J. China.

[14] Theil G., Wencker A., Kersten F., Pini G., Luecke K., Fornara P. (2013). Verification of a functionalized structured medical wire for the isolation of circulating tumor cells (CTC) in patients with renal cell carcinoma. J. Urol..

[15] Theil G., Fischer K., Weber E., Medek R., Hoda R., Lucke K., Fornara P. (2016). The Use of a New CellCollector to Isolate Circulating Tumor Cells from the Blood of Patients with Different Stages of Prostate Cancer and Clinical Outcomes—A Proof-of-Concept Study. PLoS ONE.

[16] Chudak C., Herrmann J., Lesser T. (2016). Enumeration and molecular characterization of circulating tumor cells in lung cancer patients using the GILUPI CellCollector. J. Thorac. Oncol..

[17] Dlugaszewska B., Gasiorowski L., Herold S., Nowack B., Dworacki G., Luecke K., Dyszkiewicz W. (2014). An innovative technology for in vivo isolation of circulating tumor cells in non-small cell lung cancer (NSCLC) patients and immunofluorescent detection of ALK protein. J. Thorac. Oncol..

[18] Gasiorowski L., Herold S., Morgenthaler N., Dworacki G., Luecke K., Dyszkiewicz W. (2012). A new medical device for in-vivo isolation of circulating tumor cells in non small cell lung cancer (NSCLC) patients. J. Thorac. Oncol..

[19] Gallerani G., Cocchi C., Bocchini M., Piccinini F., Fabbri F. (2017). Characterization of Tumor Cells Using a Medical Wire for Capturing Circulating Tumor Cells: A 3D Approach Based on Immunofluorescence and DNA FISH. JOVE J. Vis. Exp..

[20] Zhang H.D., Gong S.C., Liu Y.Q., Liang L.J., He S.B., Zhang Q.X., Si M.Y., Yu Z.K. (2017). Enumeration and molecular characterization of circulating tumor cell using an in vivo capture system in squamous cell carcinoma of head and neck. Chin. J. Cancer Res..

[21] Herold S., Gasiorowski L., Nowaczyk P., Schumann A., Theil G., Haubold K., Krahn T., Dyszkiewicz W., Murawa D., Lucke K. (2013). An innovative approach for in-vivo isolation of circulating tumor cells (CTCs). Eur. J. Cancer.

[22] Vermesh O., Aalipour A., Ge J., Saenz Y., Guo Y., Alam I., Park S.-m., Adelson C., Mitsutake Y., Vilches-Moure J. (2018). An intravascular magnetic wire for the high-throughput retrieval of circulating tumour cells in vivo. Nat. Biomed. Eng..

[23] Pantel K., Alix-Panabieres C. (2010). Circulating tumour cells in cancer patients: Challenges and perspectives. Trends Mol. Med..

[24] Tirino V., Desiderio V., Paino F., Papaccio G., De Rosa M. (2012). Methods for cancer stem cell detection and isolation. Methods Mol. Biol..

[25] Malheiro V.N., Spear R.L., Brooks R.A., Markaki A.E. (2011). Osteoblast and monocyte responses to 444 ferritic stainless steel intended for a Magneto-Mechanically Actuated Fibrous Scaffold. Biomaterials.

[26] Nazneen F., Galvin P., Arrigan D.W.M., Thompson M., Benvenuto P., Herzog G. (2012). Electropolishing of medical-grade stainless steel in preparation for surface nano-texturing. J. Solid State Electrochem..

[27] Rezaei B., Havakeshian E., Ensafi A.A. (2013). Stainless steel modified with an aminosilane layer and gold nanoparticles as a novel disposable substrate for impedimetric immunosensors. Biosens. Bioelectron..

[28] Somasunderam A., Thiviyanathan V., Tanaka T., Li X., Neerathilingam M., Lokesh G.L.R., Mann A., Peng Y., Ferrari M., Klostergaard J. (2010). Combinatorial Selection of DNA Thioaptamers Targeted to the HA Binding Domain of Human CD44. Biochemistry.

[29] Subramanian N., Akilandeswari B., Bhutra A., Alameen M., Vetrivel U., Khetan V., Kanwar R.K., Kanwar J.R., Krishnakumar S. (2016). Targeting CD44, ABCG2 and CD133 markers using aptamers: In silico analysis of CD133 extracellular domain 2 and its aptamer. RSC Adv..

[30] Ooji M., Stacy M., Seth M., Mugada T., Gandhi J., Puomi P. (2005). Corrosion Protection Properties of Organofunctional Silanes—An Overview. Tsinghua Sci. Technol..

[31] Collinson M.M., Higgins D.A., Kommidi R., Campbell-Rance D. (2008). Electrodeposited silicate films: Importance of supporting electrolyte. Anal. Chem..

[32] Woo H., Reucroft P.J., Jacob R.J. (1993). Electrodeposition of organofunctional silanes and its influence on structural adhesive bonding. J. Adhes. Sci. Technol..

[33] Okner R., Favaro G., Radko A., Domb A.J., Mandler D. (2010). Electrochemical codeposition of sol-gel films on stainless steel: Controlling the chemical and physical coating properties of biomedical implants. Phys. Chem. Chem. Phys..

[34] Moller R., Csaki A., Kohler J.M., Fritzsche W. (2000). DNA probes on chip surfaces studied by scanning force microscopy using specific binding of colloidal gold. Nucleic Acids Res..

[35] Gunda N.S.K., Singh M., Norman L., Kaur K., Mitra S.K. (2014). Optimization and characterization of biomolecule immobilization on silicon substrates using (3-aminopropyl)triethoxysilane (APTES) and glutaraldehyde linker. Appl. Surf. Sci..

[36] Howarter J.A., Youngblood J.P. (2006). Optimization of silica silanization by 3-aminopropyltriethoxysilane. Langmuir.

[37] Kim J., Cho J., Seidler P.M., Kurland N.E., Yadavalli V.K. (2010). Investigations of Chemical Modifications of Amino-Terminated Organic Films on Silicon Substrates and Controlled Protein Immobilization. Langmuir.

[38] Ocana C., Hayat A., Mishra R.K., Vasilescu A., del Valle M., Marty J.L. (2015). Label free aptasensor for Lysozyme detection: A comparison of the analytical performance of two aptamers. Bioelectrochemistry.

[39] Tesoro G., Wu Y.L. (1991). Silane coupling agents—The role of the organofunctional group. J. Adhes. Sci. Technol..

[40] Baumgartel T., von Borczyskowski C., Graaf H. (2013). Selective surface modification of lithographic silicon oxide nanostructures by organofunctional silanes. Beilstein J. Nanotechnol..

[41] Seguin C., McLachlan J.M., Norton P.R., Lagugne-Labarthet F. (2010). Surface modification of poly(dimethylsiloxane) for microfluidic assay applications. Appl. Surf. Sci..

[42] Xu W.J., Riikonen J., Nissinen T., Suvanto M., Rilla K., Li B.J., Wang Q., Deng F., Lehto V.P. (2012). Amine Surface Modifications and Fluorescent Labeling of Thermally Stabilized Mesoporous Silicon Nanoparticles. J. Phys. Chem. C.

[43] Heller M., Kammerer P.W., Al-Nawas B., Luszpinski M.A., Forch R., Brieger J. (2015). The effect of extracellular matrix proteins on the cellular response of HUVECS and HOBS after covalent immobilization onto titanium. J. Biomed. Mater. Res. Part A.

[44] Hosseini F., Sadjadi M., Farhadyar N. (2014). Fe_3_O_4_ Nanoparticles Modified with APTES as the Carrier for (+)-(*S*)-2-(6-methoxynaphthalen-2-yl) Propanoic Acid (Naproxen) and (RS) 2-(3-benzoylphenyl)-propionic Acid (Ketoprofen) Drug. Orient. J. Chem..

[45] Zhang Y., Gao F., Wanjala B., Li Z.Y., Cernigliaro G., Gu Z.Y. (2016). High efficiency reductive degradation of a wide range of azo dyes by SiO_2_-Co core-shell nanoparticles. Appl. Catal. B Environ..

[46] Lee S.H., Yang S.W., Park E.S., Hwang J.Y., Lee D.S. (2019). High-Performance Adhesives Based on Maleic Anhydride-g-EPDM Rubbers and Polybutene for Laminating Cast Polypropylene Film and Aluminum Foil. Coatings.

[47] Aziz M.A., Patra S., Yang H. (2008). A facile method of achieving low surface coverage of Au nanoparticles on an indium tin oxide electrode and its application to protein detection. Chem. Commun..

[48] Sun D.D., Ran Y., Wang G.J. (2017). Label-Free Detection of Cancer Biomarkers Using an In-Line Taper Fiber-Optic Interferometer and a Fiber Bragg Grating. Sensors.

[49] Jannah F., Kim J.H., Lee J.W., Kim J.M., Lee H. (2018). Immobilized Polydiacetylene Lipid Vesicles on Polydimethylsiloxane Micropillars as a Surfactin-Based Label-Free Bacterial Sensor Platform. Front. Mater..

[50] Shaimi R., Low S.C. (2016). Prolonged protein immobilization of biosensor by chemically cross-linked glutaraldehyde on mixed cellulose membrane. J. Polym. Eng..

[51] Kemmegne-Mbouguen J.C., Ngameni E., Baker P.G., Waryo T.T., Kgarebe B., Iwuoha E.I. (2014). Carcinoembryonic Antigen Immunosensor Developed with Organoclay Nanogold Composite Film. Int. J. Electrochem. Sci..

[52] Arya S.C., Agarwal N., Agarwal S. (2004). Use of polymerase chain reaction to diagnose tubercular arthritis from joint tissues and synovial fluid. Arch. Pathol. Lab. Med..

[53] Shin D.S., Kang C.K., Kim J.K., Chung W.J., Jang K.H., Lee Y.S., Ieee I. (2003). Surface modification technology for bio-MEMS. Proceedings of the Boston Transducers’03: Digest of Technical Papers.

[54] Yazdanparast S., Benvidi A., Banaei M., Nikukar H., Tezerjani M.D., Azimzadeh M. (2018). Dual-aptamer based electrochemical sandwich biosensor for MCF-7 human breast cancer cells using silver nanoparticle labels and a poly(glutamic acid)/MWNT nanocomposite. Microchim. Acta.

[55] Aghajari R., Azadbakht A. (2018). Amplified detection of streptomycin using aptamer-conjugated palladium nanoparticles decorated on chitosan-carbon nanotube. Anal. Biochem..

[56] Sefah K., Shangguan D., Xiong X., O’Donoghue M.B., Tan W. (2010). Development of DNA aptamers using Cell-SELEX. Nat. Protoc..

