# Supplementary material for: Optimizing Silanization to Functionalize Stainless Steel Wire: Towards Breast Cancer Stem Cell Isolation
Source: Materials (Basel). 2020 Aug 21;13(17):3693. doi: 10.3390/ma13173693 (PMC7504676; doi:10.3390/ma13173693)
Supplement: Supplementary file 1 [file materials-13-03693-s001.zip › materials-887222-supplementary.docx]

Supplementary Materials: Optimizing Silanization to Functionalize Stainless Steel Wire: Towards Breast Cancer Stem Cell Isolation

Aliya Bekmurzayeva ^1,2^, Kanat Dukenbayev ^3^, Helena S. Azevedo ^4^, Enrico Marsili ^3^,
Daniele Tosi ^2,3^ and Damira Kanayeva ^5,^*

^1^ Science, Engineering and Technology Program, Nazarbayev University, Nur-Sultan 010000, Kazakhstan; abekmurzayeva@nu.edu.kz

^2^ National Laboratory Astana, Nazarbayev University, Nur-Sultan 010000, Kazakhstan; daniele.tosi@nu.edu.kz

^3^ School of Engineering and Digital Sciences, Nazarbayev University, Nur-Sultan 010000, Kazakhstan; kdukenbayev@nu.edu.kz (K.D.); enrico.marsili@nu.edu.kz (E.M.)

^4^ School of Engineering and Materials Science, Queen Mary University of London, London E1 4NS, UK; h.azevedo@qmul.ac.uk

^5^ School of Sciences and Humanities, Nazarbayev University, Nur-Sultan 010000, Kazakhstan

***** Correspondence: dkanayeva@nu.edu.kz

| 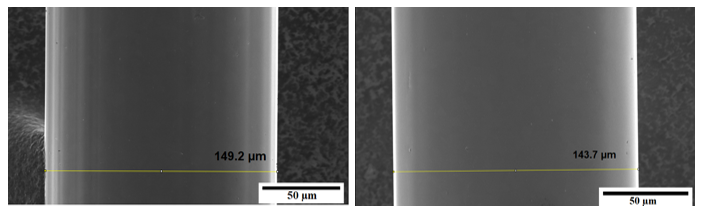 | |
| --- | --- |
| **100 s** | **200 s** |

**Figure S1.** Scanning electron micrographs of wire after electropolishing for 100 and 200 s.

| 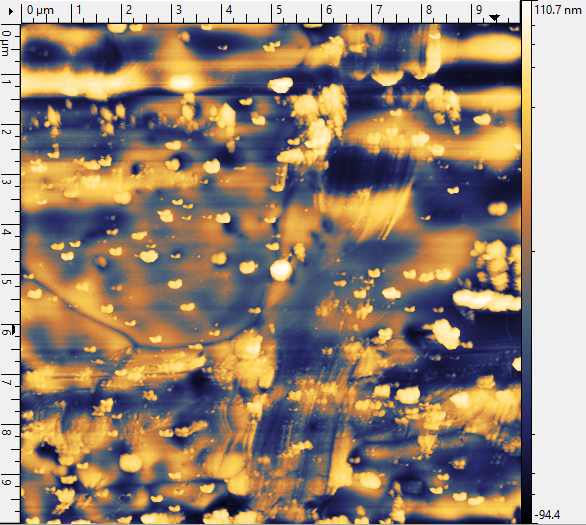 | 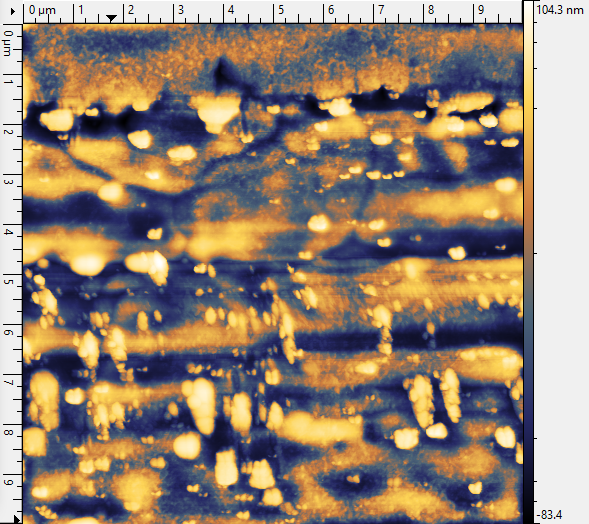 | 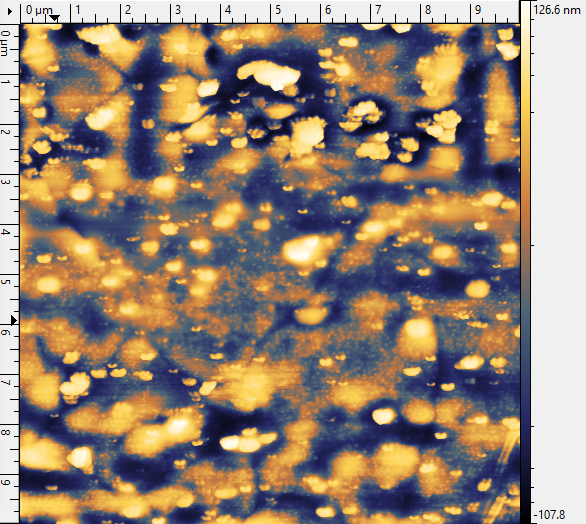 | 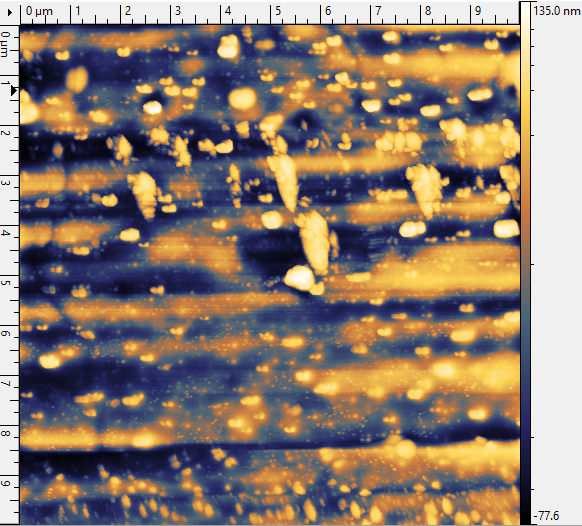 |
| --- | --- | --- | --- |
| 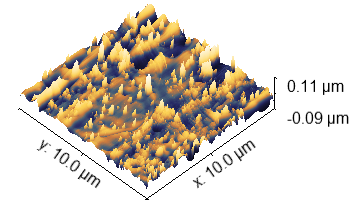 | 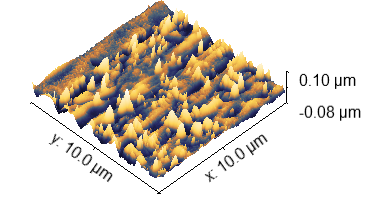 | 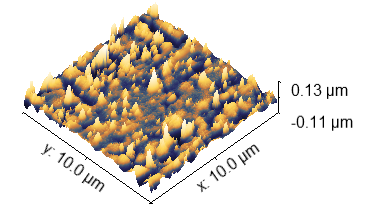 | 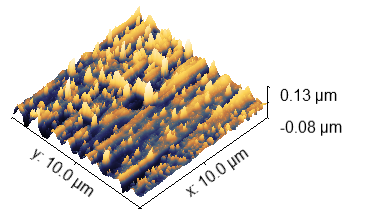 |
| **Sonic** | **-1.0** | **-0.8V** | **-0.6V** |

**Figure S2.** AFM images of APTES electrodeposition on sonicated SS (*Sonic*) wires under different potentials (10 µm × 10 µm and their corresponding 3D images).


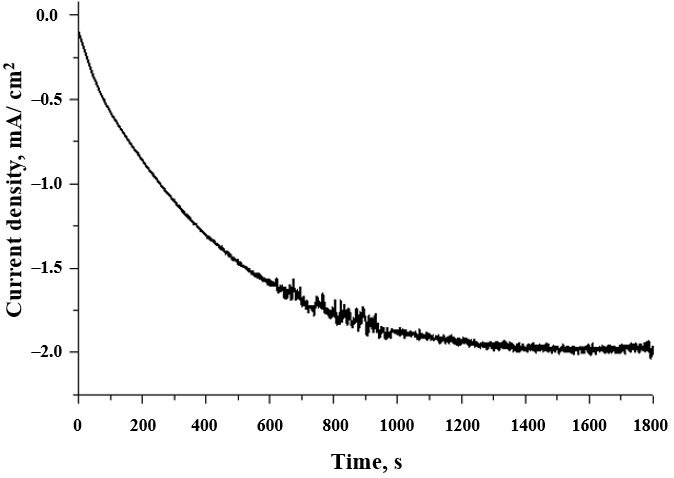


**Figure S3.** Chronoamperometry response for APTES electrodeposition on SS wires at −0.8 V for 30 min (Potential vs. QRE).


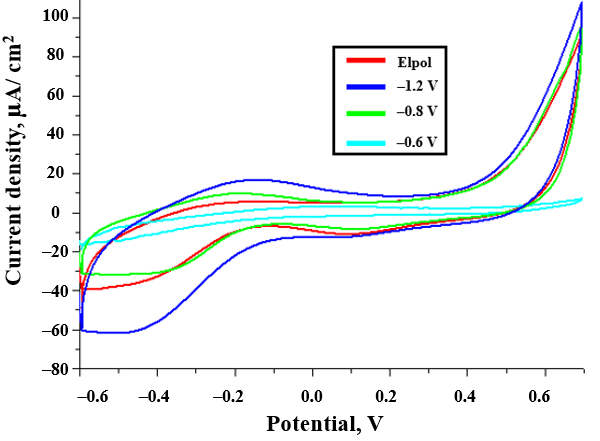


**Figure S4.** Cyclic voltammograms of electropolished SS electrode (Elpol) and silanized with APTES using different applied potentials (*HT*): −1.2; 0.8 and −0.6 V; CV done in PBS pH 7.4 containing 0.10 M KCl and 1.0 mM [Fe(CN)_6_]^3−/4−^ with a scan rate of 50 mV/s. Potential vs. RE (3 M NaCl).


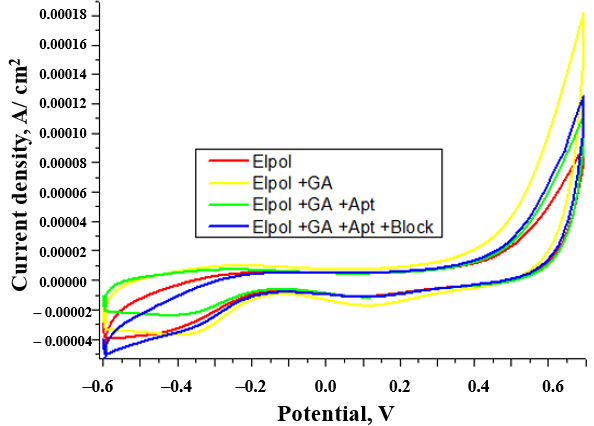


**Figure S5.** Cyclic voltammograms of functionalizing steps of a control sample (Elpol—electropolished) with aptamers. CV done in PBS pH 7.4 containing 0.10 M KCl and 1.0 mM [Fe(CN)_6_]^3−/4−^ with a scan rate of 50 mV/s. Potential vs. RE (3 M NaCl).

| 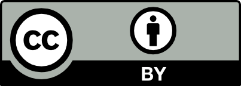 | © 2020 by the authors. Submitted for possible open access publication under the terms and conditions of the Creative Commons Attribution (CC BY) license (http://creativecommons.org/licenses/by/4.0/). |
| --- | --- |
